# eHealth-supported case management for patients with panic disorder or depression in primary care: Study protocol for a cRCT (PREMA)

**DOI:** 10.1186/s13063-019-3751-3

**Published:** 2019-12-02

**Authors:** Karoline Lukaschek, Karola Mergenthal, Dirk Heider, Alexander Hanke, Kathrein Munski, Anne Moschner, Michelle Emig, Marjan van den Akker, Antonia Zapf, Karl Wegscheider, Hans-Helmut König, Jochen Gensichen, for the PREMA-study group, K. Rupp, K. Munski, A. Moschner, M. Fellinghauer, A. Hanke, J. Gensichen, K. Lukaschek, T. Dreischulte, S. Schlüssel, H. H. König, D. Heider, K. Wegscheider, F. M. Gerlach, M. van den Akker, K. Mergenthal, M. Hanf, V. Wallraff, J. Schelling

**Affiliations:** 1 Institute of General Practice and Family Medicine, University Hospital of the Ludwig-Maximilians University of Munich, Pettenkoferstr 8a, 80336 Munich, Germany; 2 Institute of General Practice, Goethe-University, Frankfurt am Main, Germany; 3 Department of Health Economics and Health Services Research, University Medical Center Hamburg-Eppendorf, Hamburg, Germany; 4 Embloom GmbH, Bonn, Germany; 5 Techniker Krankenkasse, Hamburg, Germany; 6 Association of Statutory Health Insurance Physicians Hesse, Frankfurt am Main, Germany; 7 Department of Family Medicine, Maastricht University, Maastricht, the Netherlands; 8 Academic Centre of General Practice, Department of Public Health and Primary Care, KU Leuven, Leuven, Belgium; 9 Institute of Medical Biometry and Epidemiology, University Medical Center Hamburg-Eppendorf, Hamburg, Germany

## Abstract

**Background:**

Panic disorder (PD), frequently occurring with agoraphobia (AG), and depression are common mental disorders in primary care and associated with considerable individual and societal costs. Early detection and effective treatment of depression and PD/AG are of major importance. Cognitive behavioural exposure exercises have been shown to be effective in reducing anxiety and depressive symptoms. Practice team-based case management can improve clinical outcomes for patients with chronic diseases in primary care. The present study aims at evaluating the effects and cost-effectiveness of a primary care team-based intervention using behavioural therapy elements and case management supported by eHealth components in patients with PD/AG or depression compared to treatment as usual.

**Methods/design:**

This is a two-arm cluster-randomized, controlled trial (cRCT). General practices represent the units of randomisation. General practitioners recruit adult patients with depression and PD ± AG according to the International Classification of Diseases, version 10 (ICD-10). In the intervention group, patients receive cognitive behaviour therapy-oriented psychoeducation and instructions to self-managed exposure exercises in four manual-based appointments with the general practitioner. A trained health care assistant from the practice team delivers case management and is continuously monitoring symptoms and treatment progress in ten protocol-based telephone contacts with patients. Practice teams and patients are supported by eHealth components. In the control group, patients receive usual care from general practitioners. Outcomes are measured at baseline (T0), at follow-up after 6 months (T1), and at follow-up after 12 months (T2). The primary outcome is the mental health status of patients as measured by the Mental Health Index (MHI-5). Effect sizes of 0.2 standard deviation (SD) are regarded as relevant. Assuming a drop-out rate of 20% of practices and patients each, we aim at recruiting 1844 patients in 148 primary care practices. This corresponds to 12.5 patients on average per primary care practice. Secondary outcomes include depression and anxiety-related clinical parameters and health-economic costs.

**Discussion:**

If the intervention is more effective than treatment as usual, the three-component (cognitive behaviour therapy, case-management, eHealth) primary care-based intervention for patients suffering from PD/AG or depression could be a valuable low-threshold option that benefits patients and primary care practice teams.

**Trial registration:**

German clinical trials register, DRKS00016622. Registered on February 22nd, 2019.

## Background

Panic disorder (PD), a type of anxiety disorder, is a severe and persistent mental disorder associated with a high degree of subjective distress and occupational and social disability [1]. PD frequently occurs with agoraphobia, defined as anxiety about being in places or situations from which escape might be difficult or embarrassing or in which help might not be available in the case that escape is needed [2]. Depression is one of the most commonly occurring mental disorders [3] and is associated with considerable individual and societal costs [4, 5]. Major depression is highly associated with panic disorder (OR 29.4; 95% CI 19.9–43.4) [6]. Early detection and effective treatment of depression and panic disorder are thus of major importance.

In primary care, depression occurs in 5–10% [7] of patients, panic disorder in about 7% [8]. Evidence-based guidelines have been established for the diagnosis and optimal management of depression [9] and anxiety disorder [10, 11]. Nevertheless, not all patients receive adequate treatment yet [12].

Primary care physicians (PCPs) play a key role in the care of patients with depression and panic disorder with or without agoraphobia (PD/AG) [8, 13]. They are usually the point of first contact with the healthcare system and can detect depression or anxiety disorders at an early stage, can initiate treatment, or refer the patient to a specialist. The primary care setting includes (1) first-contact care and gatekeepers; (2) longitudinality and managed care; (3) comprehensiveness and benefit packages; and (4) coordination of the referral process [14].

Electronic health (eHealth) technologies represent one strategy for improving the accuracy and completeness of clinical information collected from patients. These technologies can be used to gather, manage, and disseminate health information via computers, tablets, and mobile devices. eHealth technologies can support clinical practice by facilitating the accessibility of patient data and appropriate evidence-based guidelines, offering a potential strategy for improving the safety, quality, and efficiency of care [15].

### Evidence-based treatments for PD/AG and depression in primary care

Both psychological and pharmacological interventions are recommended in the treatment of PD/AG [11] and depression [9]. Cognitive behavioural therapy (CBT) is considered at least equally effective in the treatment of PD/AG as pharmacotherapy and can result in better long-term effects [16, 17]. PCPs can deliver key elements of CBT, e.g. psychoeducation, exposure exercises, and relapse prevention, as a first step in treatment [18, 19].

In the intervention arm of our study (PREMA), patients work through a standardized psychological treatment protocol including CBT elements [20–23]. Small effect sizes were found for treatments that were delivered by primary care therapists not specialized in mental health [24]; regarding depression and anxiety, treatments including CBT delivered by primary care therapists are potentially more effective than usual care [25].

Collaborative care is associated with significant improvement in depression and anxiety outcomes compared with usual care [26]. Primary care-based case management led by a medical assistant (MA) may be a key ingredient of effective collaborative care [27]. Interventions including CBT elements and MA-led case management were effective in the treatment of primary care patients suffering from PD/AG [28] or depression [29].

Currently, patients in Germany with PD/AG or depression wait for several months for an available psychotherapeutic treatment [30]. Thus, a low-threshold treatment including elements of CBT and case-management supported by eHealth components and adapted to primary care settings could bridge the waiting time. A cluster randomized controlled two-arm study including 74 primary care practices with 626 patients aged 18 to 80 years with major depression (PROMPT trial) indicated that case management provided by primary care practice-based MA may reduce depression symptoms more than usual care: compared with control patients, intervention recipients had lower mean PHQ-9 values in depression symptoms (− 1.41 [95% CI, − 2.49 to − 0.33]; *p* = 0.014) after 12 months [29]. Another randomized controlled two-arm study including 419 primary care patients (mean age 46.2 ± 14.4 years) with PD/AG (PARADISE trial) showed that a team-based exercise program combined with case management can improve symptoms of anxiety to a greater extent than standard primary-care treatment (*p* = 0.008). The intergroup difference in the reduction of the BAI score (range 0–63) was 4.0 points [− 6.9; − 1.2] at 12 months [28].

### Aims and objectives

Based on the PROMPT and PARADISE trials, we designed the PREMA trial. The aim of the PREMA (“eHealth supported case management for mentally ill patients in primary care”) study was to evaluate the effects and cost-effectiveness of a primary care team-based intervention using behavioural therapy elements and case management supported by eHealth components in patients with PD/AG or depression compared to treatment as usual (standardized TAU). It is hypothesized that this intervention results in a significantly greater improvement of symptoms of PD/AG or depression than standardized TAU (primary objective). Secondary objectives are to determine if the programme is superior to usual care regarding further clinical parameters, patients’ perspectives on receipt of care, and direct and indirect health-economic costs.

## Methods/design

### Trial design and setting

PREMA is a two-arm cluster-randomized, controlled trial (cRCT). The trial will be conducted in primary care practices in Hesse, Germany. Randomization will be performed at cluster level (cluster = primary care practice).

Participating primary care practices will recruit patients within a 3-month screening phase. After that, the primary care practices (and therefore all of the respective recruited patients) will be allocated to either the intervention arm of the study (PREMA exercises) or the control-arm (treatment as usual, TAU) using a randomization list generated by the UKE (see below).

In the intervention group (IG), patients receive case management and training supported by primary care practice teams and eHealth components over 12 months. In the control group (CG), patients receive standardized TAU.

With PREMA, we follow the guidance to researchers on the process for developing and evaluating interventions provided by the Medical Research Council (MRC) guidelines [31] for complex interventions to the greatest possible extent. Thus, we ensure that (a) the intervention is empirically and theoretically founded and (b) considerations are given to the effectiveness of the intervention and how it might work.

### Target population and eligibility criteria@bhan

#### Inclusion and exclusion criteria for Primary care physicians

Inclusion criteria for participating PCPs are:

- The physician must be registered in the German statutory healthcare system as a primary care physician (“Kassenzulassung”)
- The physician must have a qualification in basic psychosomatic care (Psychosomatische Grundversorgung, Bundesärztekammer, 2001) [32] to ensure that they can provide a basic level of mental health care and to ensure patient safety
- Primary care practice in the Federal State of Hesse, Germany
- Practice team includes at least one medical assistant (MA) with ≥ 3 years work experience

Exclusion criteria for participating PCPs are:

- Practice offers private medical treatment only

### Recruitment of primary care practices

Recruitment of primary care practices will be organized by the Association of Statutory Health Insurance Physicians Hesse (Kassenärztliche Vereinigung Hessen, KVH). The KVH also checks whether the conditions of participation have been fulfilled on the basis of the contract § 140a SGB V.

#### Inclusion and exclusion criteria for patients

Patients must meet the following inclusion criteria to be eligible for enrolment into the trial at baseline: suffering from panic disorder ± agoraphobia (ICD-10 F41.0, F40.01) and/or depression (ICD-10 F32–34) and being treated in a primary care practice in Hesse (cut offs PHQ-9 ≥ 9 and ≤ 22; OASIS ≥ 8); being able to provide written informed consent; holding a participating health insurance policy; age ≥ 18 years; sufficient German language skills to follow instructions; internet and telephone access at home.

Patients are excluded from enrolment if any of the following exclusion criteria apply: known psychosis; acute suicidality; concomitant therapy: panic- or depression-specific psychotherapy at baseline; patient unsuitable for intervention (according to PCP’s assessment).

### Screening and recruitment of patients

**Fig. 1:**
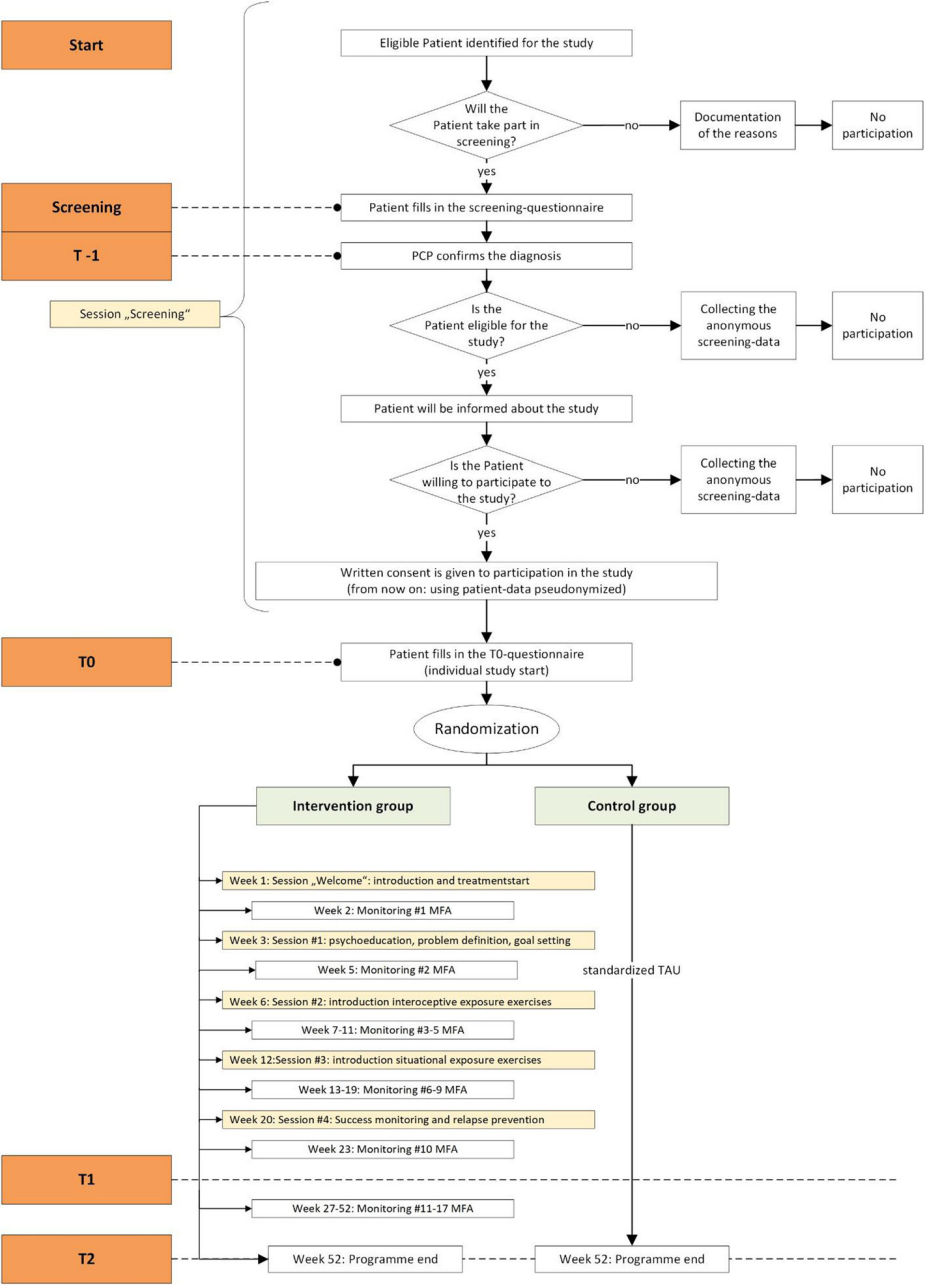
Flow chart of the PREMA study

Figure 1 is a flow chart of the study. This protocol follows the “Guidance of Standard Protocol Items: Recommendations for Interventional Trials (SPIRIT) 2013 statement” [33] Additional file 1.

In the screening phase, which lasts up to 3 months, MAs compile a list of patients which seem to be eligible for the PREMA trail. If such a patient presents him/herself at the practice, he/she is checked for inclusion criteria and informed about the PREMA trial. Additionally, patients who are identified as eligible during normal practice opening hours can be recruited. After including 12–13 patients, or after 3 months at the latest, primary care practices are allocated to either the intervention or the control arm. Patients are screened for depression or panic disorder using the Patient Health Questionnaire (PHQ)-9 [34, 35] and the Overall Anxiety Severity and Impairment Scale (OASIS) [36, 37]:, cut offs PHQ-9 ≥ 9 and ≤ 22; OASIS ≥ 8. If a patient is screened positively for PD/AG or depression, the PCP confirms the diagnosis through the diagnostic interview. After that, the patient is informed about the study details. Written informed consent is obtained from all participants.

### Informed consent procedures

Prior to enrolment, after the eligibility of a patient has been checked and confirmed during screening before the baseline assessment at T0, patients willing to participate in the study will be provided with a full explanation of the trial verbally and in writing (patient information sheet). Written consent will be obtained before any trial-specific procedures commence. All participants may withdraw their informed consent from the trial at any time and without any negative consequences for further treatment.

### Randomization

Randomization will be performed at cluster level (cluster = primary care practice) after a practice has successfully included 12–13 patients, or after the 3-month screening process at the latest.

The randomization list with randomly varying block lengths will be generated by an independent person affiliated to the Institute of Medical Biometry and Epidemiology, UKE, Hamburg-Eppendorf, based on computer-generated sequences and stratified for practice type (urban vs rural). The randomization list is deposited at the online platform of our eHealth partner, Embloom. Embloom unites the practice list provided by the KV with the randomization list provided by the UKE and generates the randomization results.

### Basic training for all participating primary care practices

All participating PCPs will receive general information and online material regarding diagnosis, therapy, and treatment of PD/AG and depression. The provided material is based on the national guidelines for depression [11] and for anxiety disorders [9]. Moreover, PCPs will be schooled in study procedures and the use of the TelePsy online platform.

All participating MAs will be schooled in the use of the online platform, study procedures, the screening process, and the recruitment and inclusion of patients. They will receive general information and online material on panic disorder and depression.

All online material is provided on the Embloom platform.

### Training for primary care practices allocated to the intervention group

Primary care practices allocated to the IG are additionally trained in treating patients by use of a practice team-supported, self-managed exposure programme. This online training comprises the following contents: (1) rationale of exposure techniques with regard to PD/AG and depression; (2) treatment plan; (3) structure of self-help materials; (4) practice team collaboration and case management; (5) only for GPs—analysing patients’ feared stimuli or avoidance/withdrawal behaviour, planning and implementing individually appropriate exposure exercises or pleasure aims in co-operation with the patient, supervising treatment progress and evaluating success of exposure exercises and pleasure aims, relevant interactions of psycho-pharmacological treatments; (6) only for MAs—conducting telephone contact with patients with the help of a monitoring checklist.

Both the PCPs and the MAs receive detailed online treatment manuals provided on the online platform.

### Intervention group

Based on the PROMPT [29] and PARADISE [28] trials, we designed the intervention of the PREMA trial as a low-threshold intervention containing the three elements CBT, case management, and eHealth-support for patients suffering from AG/PD or depression.

After randomization, primary care practices in the IG will receive online training material (therapy manuals for PCPs and MAs, intervention videos). Additionally, patients in the IG will receive online information about their disorder and trial procedures. Treatment in the IG consists of four face-to-face sessions of approximately 30 min delivered by the PCP and practice-based case management delivered by the PCP and the MA.

The four sessions delivered by the PCP include the following:

- Session 1 (week 3 after treatment start): Psycho-education on depression or panic disorder. Patients learn about the symptoms and the theoretical background of their disorder as well as the treatment procedure.
- Session 2 (week 6): Interoceptive exposure exercises. The PCP introduces exercises that provoke the patient’s typical physical reaction. Patients learn to conquer their initial fears and to find promising solutions (activation and motivation of the patients).
- Session 3 (week 12): Situational exposure exercises. The patients are confronted with situations that trigger their fear or from which they withdraw. Patients with depression aim to experience pleasure in daily life activities.
- Session 4 (week 20): Success monitoring and relapse prevention.

Throughout the sessions, the patients are encouraged to note questions, successes, or failures online on the eHealth platform.

Case management by the MA consists of 17 telephone calls (approximately 10 min each), in which the MA asks about the patient’s well-being, completes the monitoring checklists (JAMoL [38] for patients with PD/AG, DeMoL [39] for Patients with depression), and motivates/encourages the patients. Critical responses should prompt the MA to inform the attending PCP immediately. Questionnaire responses are noted online on the eHealth platform.

### Control group

Patients allocated to the CG will receive standardized TAU: Treatment will be based on current German recommendations for the diagnosis and treatment of depression [9] and anxiety disorders [11]. The PCP has access to general information about diagnosis and therapy of panic disorder and depression provided via the online platform. In a basic training, PCPs will receive online training material and medical information about depression and panic disorder, based on the current national guideline for depression [9] and anxiety disorders [11]. Upon completion of the study, the online material of the IG primary care practices will be made available for CG primary care practices.

### Study outcomes

#### Primary outcome

To evaluate the mental health (depression and panic disorder) of trial participants, the Mental Health Index-5 (MHI-5) score [40] will be applied. The MHI-5 is a five item subscale of the Short Form (36) Health Survey (SF 36) [41]. Each question is answered on a five-point Likert scale (from 1 not at all to 5 more than five times per week/severe; total range 5–25). Higher score indicates better mental health. Using a standard linear transformation, the score will be transformed to a range of 0–100. Internal consistency (Cronbachs α) ranges from 0.67 to 0.95 [42]. The MHI-5 was validated in a primary care setting [42]; ROC analyses indicated that a cut-off score of 23 on the MHI-5 yielded a sensitivity of 91% and a specificity of 58% for predicting provisional diagnoses of major depression or panic disorder. The MHI-5 will be assessed online at baseline (T0) and 6 (T1) and 12 (T2) months after baseline.

#### Secondary outcomes

Secondary outcomes include: depression measured using the Patient Health Questionnaire (PHQ-9) [43]; anxiety measured using Overall Anxiety Severity and Impairment Scale (OASIS) [37]; number and severity of panic attacks, measured using two items (A1, A2) of the panic and agoraphobia scale (PAS) [44]; agoraphobic avoidance behaviour, measured using the mobility inventory (MI) [45], “alone “subscale; patient evaluation of the medical care received, measured using the Patient Assessment of Chronic Illness Care (PACIC) [46]; adherence for medication ([47]), days free of depression (DFD) [48], days free of anxiety (AFD) [49], health-related quality of life as measured by the EuroQol-questionnaire (EQ-5D) [50].

Secondary outcomes from secondary data include: health service use (number of inpatient hospitalizations, days of inpatient hospitalizations, outpatient medical treatment, medication), health care costs (costs of inpatient hospital care, inpatient rehabilitation, outpatient services and primary care, medical supplies and medication, sick pay costs, and total health care costs), sick leave days and comorbidities (Elixhauser-Index [51]).

Measurements will be performed at baseline (T0), at 6-month follow-up (T1), and at 12-month follow-up (T2).

### Study procedures and timing schedule

The baseline assessment (T0) takes place by online self-reported questionnaires.

In the IG, the MA’s first telephone contact with the patient is scheduled for week 2 after the primary care practice is allocated to the IG; the PREMA sessions with the PCP start in week 3. In the CG, patients receive TAU.

The primary outcome (MHI-5) will be assessed by self-reported online questionnaires at baseline (T0) and 6 months (T1) and 12 months (T2) after baseline. Secondary outcomes will be assessed at baseline (T0) and 6 months (T1) and 12 months (T2) after baseline. The initial screening measures for depression (PHQ-9) and panic disorder (OASIS) will be repeated at T1 and T2.

For a detailed description of study activities and the components of the intervention, see Fig. 1 and Table 1.

**Table 1:** Outcome parameters

Indicator	Instrument	Time point				Data source
		T_−1_	T_0_	T_1_	T_2_	
Screening and diagnosis	OASIS, PHQ-9, ICD-10 checklist	x				Primary care physician/diagnostic interview
Mental health (depression and panic disorder)	Mental Health Index-5 (MHI-5)		x	x	x	Patient assessment via questionnaire
Depression level	PHQ-9			x	x	Patient assessment via questionnaire
Anxiety level	OASIS			x	x	Patient assessment via questionnaire
Severity of panic attacks	Panic and Agoraphobia scale (PAS)		x	x	x	Patient assessment via questionnaire
Avoidance behaviour	Mobility Inventory for Agoraphobia (MIA)		x	x	x	Patient assessment via questionnaire
Quality of care	Patient Assessment of Chronic Illness Care (PACIC)		x	x	x	Patient assessment via questionnaire
Medication adherence	Adherence score		x	x	x	Patient assessment via questionnaire
Quality of life	EQ-5D-5 L index		x	x	x	Patient assessment via questionnaire
Comorbidity	Elixhauser index		x	x	x	Routine data
Health service use	Number of inpatient hospitalizations, days of inpatient hospitalizations, outpatient medical treatment, medication (defined daily dose, DDD)		x	x	x	Routine data
Sick leave	Days of inability to work, sickness benefit, rehab		x	x	x	Routine data
Health care costs	Inpatient hospital care costs, inpatient rehabilitation costs, outpatient (ambulatory) services and primary care costs, costs for medical supplies, costs for drugs, sick pay costs, total health care costs		x	x	x	Routine data
Acceptance, attitude, expectations, feasibility, training, communication, implementation, work satisfaction	Ad hoc items		x	x	x	Primary data

The end of the clinical trial is defined by the last individual trial-specific examination of the last patient who is still participating in the trial.

### Participation discontinuation

If a patient withdraws his/her written informed consent, the assigned study intervention will be discontinued for him/her. Severe adverse events (SAEs) are defined as a patient’s death, life-threatening event, clinically relevant severe deterioration of depression, or anxiety symptoms, acute suicidality, or adverse events that would constitute an unacceptable risk for the patient. All SAEs will be documented by the PCP and evaluated by the PI and the Data and Safety Monitoring Board (DSMB) at the principle investigator’s (PI’s) discretion to ensure safety evaluations follow the four-eye principle. If a patient completely drops out of the study, a final assessment, especially of the primary outcome, should be conducted.

### Accompanying studies

#### Health economic evaluation

Health economic evaluation includes the analysis of health service use, health care costs, as well as cost-effectiveness. Evaluation of health care costs will be conducted from the perspective of the health insurance fund and will be based on secondary data. To analyze cost-effectiveness the incremental cost effectiveness ratio (ICER) will be calculated, which is the ratio of the difference in mean health care costs and quality-adjusted life years (QALYs) between IG and CG after 12 months of intervention. QALYs be will calculated based on health-related quality of life measured with the EQ-5D-5 L-Index (primary data) and its German value set developed by Ludwig et al. [52].

#### Process evaluation

Comprehensive qualitative-quantitative evaluation is used to depict the processes involved in implementing primary care-based case management [53], whereby the qualitative analysis has priority.

The qualitative part includes the evaluation of communication and collaboration among participants, feasibility, and factors that support and inhibit the implementation of eHealth-supported primary care-based case management in patients, PCP, and MA.

For the quantitative part, participants (patients, PCP, MA) will be required to fill in a questionnaire at the beginning of the study on their acceptance of novel therapies and their attitudes towards and expectations of eHealth-supported therapy. After 6 months, the participants will evaluate the training, which was carried out using the online platform. At the end of the study (after 12 months), a survey will be conducted on various aspects concerning the implementation of the new treatment, including the degree of utilization (proportion of patients who received which therapy and how often), feasibility (protocol-compliant implementation), and saturation. Using data from the Embloom platform's initial use, continued use and reach of the program will be analyzed.

### Materials and methods

The recruitment for the qualitative interviews will be a random sample among study participants. Qualitative interviews and focus group discussions will be conducted using a semi-structured interview guideline. The guideline will be developed, piloted, and adapted on the basis of a literature search. Individual interviews will take place with about 20–30 patients, and expert interviews with approximately 10–15 PCPs and 10–15 MAs from the IG. Three-to-five focus group discussions will be held with 15–20 PCPs and 15–20 MAs (mono- and interdisciplinary) at the end of the study. The data will then be transcribed verbatim and the transcripts checked for accuracy. Participants’ names and further details that could be used for identification will be changed. With the support of commonly used software programs (e.g. MAXQDA), transcripts will be analyzed using qualitative content analysis.

For the quantitative part, self-developed questionnaires will be filled in by patients, PCPs, and MAs. To develop the questionnaire, a literature review was carried out and the results checked to ensure they are in line with the aims of the PREMA study. The questionnaire will then be adapted and pilot tested again. Commonly used software programs (e.g. SPSS) will be employed to conduct descriptive and regression analysis of the primary data. Triangulation of the qualitative and quantitative data will be performed if possible.

### Statistical planning and analysis

#### Power considerations and sample size calculation

Sample size calculation is based on power considerations regarding the statistical difference between the Intervention (PREMA) and treatment as usual (TAU). In a primary care setting, effect sizes of 0.2 standard deviation (SD) on the primary outcome were regarded as relevant. This effect size was the basis for our sample size calculation. Assuming a desired power of 0.9, a type 1 error of 0.05 (two-sided) and a correlation of the baseline values with the primary outcome of 0.5, a sample size of 395 patients per study arm would be necessary. Considering the hierarchical data structure defined by the cluster randomization, an average cluster size of ten patients per primary care practice and an intra-cluster-correlation coefficient (ICC) of 0.05, the design effect would be 1.45. Therefore, the necessary sample size per study arm increases to 590 patients in 59 primary care practices (in total, 1180 patients, 118 practices). Assuming a drop-out rate of 20% of practices and patients, we aim at recruiting 1844 patients in 148 primary care practices. This corresponds to 12.5 patients on average per primary care practice.

#### Statistical analysis for primary and secondary outcomes

Baseline characteristics of primary care practices and patients will be described using relevant descriptive statistics. The primary analysis follows the “intention-to-treat” principle and will be based on all available data of all included patients. Main outcome is the Mental Health Index 5 (MHI 5) score at baseline (T0) and 6 (T1) and 12 (T2) months after.

The score will be analysed as differences to baseline. For the analysis of the intervention effect, a linear mixed model (LMM), taking the hierarchical data structure into account, will be adapted. The patients and the GP practices they are nested in are included in the LMM as random factors; the practice location (urban versus rural), the baseline value of the score, as well as the treatment group and the time of measurement are included as fixed factors. Fixed-effects estimators are reported with 95% confidence intervals. If necessary, a sensitivity analysis with multiple imputation of missing values will be conducted.

The secondary outcomes will also be described using relevant descriptive statistics. Continuous variables will be analysed with LMMs and for dichotomous outcomes mixed logistic regression models will be performed. The other model parameters will be set as in the primary endpoint analysis.

The analyses will be performed using R (3.4.4 or newer) or Stata (14.2 or newer). All tests will be two-sided with alpha = 0.05.

A more detailed description of all analyses will be found in the statistical analysis plan, which will be written prior to the commencement of the analyses. The final definition of the statistical models will be performed after a blind review of the study data.

For the health economic evaluation, differences in mean health care costs between IG and CG will also be analysed by implementing linear mixed regression models to consider for several time points per respondent and for the underlying cluster structure. Thereby, non-parametric bootstrapping will allow calculation of unbiased standard errors despite the well-known skewness of health care cost.

For analysing the statistical uncertainty of the ICER, net benefit regression will be implemented in order to construct cost-effectiveness acceptability curves (CEAC).

### Data management

Administrative forms will be collected at KVH (participation forms of the practices) and the health insurer (patient’s participation forms and copies of the informed consents). The online platform will collect data online at each measurement point (T−1, T0, T1, T2). Patient’s medical data will be transferred to a study database in pseudonymized form. Mistakes and errors will be corrected by corresponding form, queries in the practices, or directly with the patient. The data manager will secure the study database and approves its completeness. Pseudonymized data will be transferred to cooperating scientific institutes.

### Data collection and transmission

The study data stem from patients in general practices in Hesse who fulfil the inclusion criteria. Data transmissions between the project partners works as follows.

Primary data will be collected on the Embloom online platform. Embloom will transmit the data together with the corresponding record ID to the trust authority. Organizational and secondary data (patient data from the general practice, participation forms, and informed consents) will be collected as well. Patient’s participation forms will be merged with the corresponding Embloom record number in the practices and sent to the health insurer. Additionally, the practices will send their participation forms for the medical care contract and billing data to KVH, which will generate a list of practices from the participation forms and send it to Embloom. The practice’s billing data will be sent from KVH to the health insurer, which will create a key list with a unique identifier as well as the Embloom record number. This list will be sent to the trust authority together with the routine data (including billing data). The trust authority will merge routine and primary data, pseudonymize it, and send it to the scientific institutions which will evaluate the data.

For randomization, UKE will send a randomization sequence to Embloom, which will merge the list of practices from KVH and the randomization sequence to generate the randomization result. The randomization result will then be sent to KVH as well as the participating practices.

### Data handling

#### Data safety and monitoring board

An independent DSMB has been established to monitor the course of the study, recruitment, patient safety, the integrity of the trial, and, if necessary, to give a recommendation to the coordinating investigator and sponsor for discontinuation, modification, or continuation of the study. The DSMB will confer twice a year. Furthermore, the DSMB will periodically review the safety-relevant events reported to this board. The members of the DSMB are Prof. Dr Matthias Berking (Erlangen), Prof. Dr Karl-Jürgen Bär (Jena), Prof. Dr André Scherag (Jena).

## Discussion

The aim of the PREMA trial is to evaluate the effect of a three-component (CBT, case-management, eHealth) primary-care-based intervention for patients suffering from PD/AG or depression.

A limitation of the study might be the diagnosis-unspecific generic primary outcome, the MHI-5. The briefness of the instrument (five items) entails a limited, yet acceptable, validity of the measured effects. A selection bias of participating PCPs and patients may limit the generalisability of the results. Even though the applied PREMA is adapted to the primary care setting, there may still be barriers to implementation in daily clinical practice, e.g. due to limited resources in PCP practices. In Germany, 87.7% (*n* = 72.8 million) of all German residents had a statutory health insurance (Gesetzliche Krankenversicherung, GKV) in 2018. Until now, however, the study takes place in only one federal state (Hesse), and only one health insurer (Techniker Krankenkasse, TK) participates in this study. Of those Hessians with a statutory health insurance, about 17.5% have TK insurance. This setting limits the generalisability of our findings and might also lead to insufficient recruitment, a major risk in the execution of the study. Thus, we reserve the right to include other health insurers or expand to other federal states. Finally, we did not conduct a systematic pilot study; however, we draw our experiences from other successful similar designed studies (PARADIES, PROMPT). Based on these studies, we designed PREMA as the next step towards a broader implementation strategy; thus, we are able to estimate the recruiting potential from experiences made in these previous studies [28, 54].

### Trial status

At the time of manuscript submission, the study has been approved by the ethics committee of the Goethe-University, Frankfurt/Main, Germany. We expect enrolment of first patient in late summer 2019.

### Protocol version

Version 01/20190730.

## Supplementary information

**Additional file 1.** SPIRIT 2013 Checklist: Recommended items to address in a clinical trial protocol and related documents*.

## Data Availability

Data sharing is not applicable to this article as no datasets were generated or analyzed during the current study (study protocol). When the trial is completed, primary data of the study will be available on request only.

## Abbreviations

AG: Agoraphobia
CBT: Cognitive behavioural therapy
CG: Control group
cRCT: Cluster-randomized, controlled trial
DSMB: Data safety and monitoring board
eHealth: Electronic health
ICD: International Classification of Diseases
IG: Intervention group
MA: Medical assistant
MHI-5: Mental Heath Index 5
OASIS: Overall Anxiety Severity and Impairment Scale
PCP: Primary care physician
PD: Panic disorder
PHQ: Patient Health Questionnaire
PI: Principle investigator
ROC curve: Receiver operating characteristic curve
SAE: Severe adverse event
SD: Standard deviation
TAU: Treatment as usual
